# Sulfur‐Doped Carbon with Enlarged Interlayer Distance as a High‐Performance Anode Material for Sodium‐Ion Batteries

**DOI:** 10.1002/advs.201500195

**Published:** 2015-08-25

**Authors:** Long Qie, Weimin Chen, Xiaoqin Xiong, Chenchen Hu, Feng Zou, Pei Hu, Yunhui Huang

**Affiliations:** ^1^ State Key Laboratory of Material Processing and Die & Mould TechnologySchool of Materials Science and EngineeringHuazhong University of Science and TechnologyWuhanHubei430074China

**Keywords:** anodes, electrochemical performance, S‐doped carbon, sodium‐ion batteries

## Abstract

**S‐doped carbon is investigated as a high‐performance anode material** for sodium‐ion batteries. Due to the introduction of a high‐content of S atoms, the as‐obtained S‐doped carbon shows an enlarged interlayer distance. As an anode, a high specific capacity of up to 303 mAh g^−1^ is achieved, even after 700 cycles at 0.5 A g^−1^.

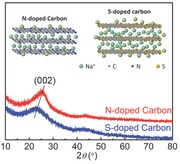

The increasing demand on lithium‐ion batteries (LIBs) has led to potential insufficiency of lithium resource. To address this concern, more and more efforts have been made to explore cost‐effective rechargeable battery systems beyond LIBs.^1^ For large‐scale stationary applications where the gravimetric energy density is not the most critical concern, room‐temperature sodium‐ion batteries (SIBs) have been regarded as the prime candidates due to the inexhaustible Na resource and the low price of Na raw materials.^2^

Since Na^+^ ion has larger ionic radius and inappropriate insertion/extraction potential as compared with Li^+^ ion, many electrode materials used for LIBs show very limited activity or even fail when being tested as hosts for Na^+^ ions, which greatly hinders the development of SIBs. To promote the practical application of the SIB technology, it is urgent to explore new host materials for Na^+^ ions. A number of transition metal oxides, poly‐anionic, and Prussian blue compounds have been identified as promising cathode materials for SIBs.^3^ Meanwhile, the choices for anodes seem limited:^4^ only a few materials such as disordered carbons, Ti‐based intercalation compounds, a few metal alloys, oxides, and sulfides have displayed satisfactory Na^+^‐ion storage capacities, among which low crystalline hard carbons are the most attractive ones for commercial application of SIBs due to their low price.

In light of the successful strategies of carbonaceous anodes for LIBs, many researchers tried to find carbons with high Na^+^‐ion storage capacities by reducing their particle size. Carbons with various nanostructures such as hollow nanospheres,^5^ nanosheets,^6^ nanofibers,^7^ hollow nanowires,^8^ graphene,^9^ and porous carbons 10 have been investigated, and reversible capacities of ≈300 mAh g^−1^ have been achieved.^11^ However, the reduced particle sizes or porous structures always cause enlarged specific surface area of the carbons, leading to low initial Coulombic efficiency, and thus greatly reduce the energy density of the battery and result in poor cycling performance when being used in full cell configurations.^12^ In this way, reducing materials to nanosizes does not seem as a thorough solution to high‐performance carbonaceous anodes for SIBs.

On the other hand, the Na^+^‐ion storage mechanism in carbonaceous hosts, which was proposed as “house of cards” model by Dahn and co‐workers, is similar to that of Li^+^‐ion storage.^13^ To facilitate the insertion–extraction of Na^+^ ions, large interlayer distance is needed.^14^ Based on experimental results and theoretical calculations, Cao et al. concluded that carbon materials with interlayer distances of >0.37 nm could act as Na^+^‐ion insertion anodes.^8^ An expanded graphite with enlarged interlayer lattice distance of 4.3 Å was fabricated by Wen et al. through a two‐step oxidation–reduction process and showed excellent cycling stability, making it a competent anode material for SIBs.^15^

Heteroatom doping is a widely used approach to tune the physicochemical properties of carbon materials.^16^ Nitrogen doping has been recognized as an effective route to improve the Na^+^‐ion storage capacities of carbons.^8^, ^17^ Compared to N‐doped carbon (NC), sulfur‐doped carbon (SC) seems to be more suitable as anode for SIBs because the covalent radius of S (102 pm) is much larger than those of C (77 pm) and N (75 pm).^18^ Introduction of S atoms can significantly enlarge the interlayer distances of carbons,^19^ which should be able to facilitate the insertion–extraction of Na^+^ ions.

To validate the supposition, herein, for the first time, we investigated the Na^+^‐ion storage performances of SC (S content: 15.17 wt%). For comparison, NC (N content: 12.14 wt%) was also prepared and investigated under same conditions. The results show that the as‐obtained bulk‐sized SC possesses larger interlayer distance (≈0.39 nm) but lower specific surface area (39.8 m^2^ g^−1^) than those of NC with nanosheet structure (≈0.36 nm and 139.7 m^2^ g^−1^). Benefitting from the larger interlayer distance and the lower specific surface area, SC delivers a high reversible specific capacity of 482.1 mAh g^−1^ with an initial Coulombic efficiency of 73.6% at a current density of 0.1 A g^−1^, and a high‐capacity retention of 303.2 mAh g^−1^ even after 700 cycles at 0.5 A g^−1^ as anode for SIBs, much higher than those of NC.

The precursor for SC, poly(3,4‐ethylenedioxythiophene) (PEDOT), was synthesized by chemical oxidation polymerization of 3,4‐ethylenedioxythiophene (EDOT, C_6_H_6_O_2_S) at room temperature. The scanning electron microscopy (SEM) image (Figure S1a, Supporting Information) reveals that the as‐prepared PEDOT consists of bulk particles with sizes in the range of 0.5−2 μm. In contrast, polypyrrole (PPy), the precursor for NC, was prepared via chemical oxidation polymerization of pyrrole (Py, C_4_H_5_N); SEM image (Figure S1b, Supporting Information) shows a nanosheet‐like morphology with a thickness of <50 nm, which is totally different from PEDOT prepared under the same condition. The difference in their morphologies can be attributed to the different physicochemical properties between PEDOT and PPy. PEDOT and PPy as the precursors were then carbonized at 700 °C for 2 h under an argon atmosphere, leading to the formation of SC and NC with same microstructures (**Figure** **1**a and Figure S2a in the Supporting Information).

**Figure 1:**
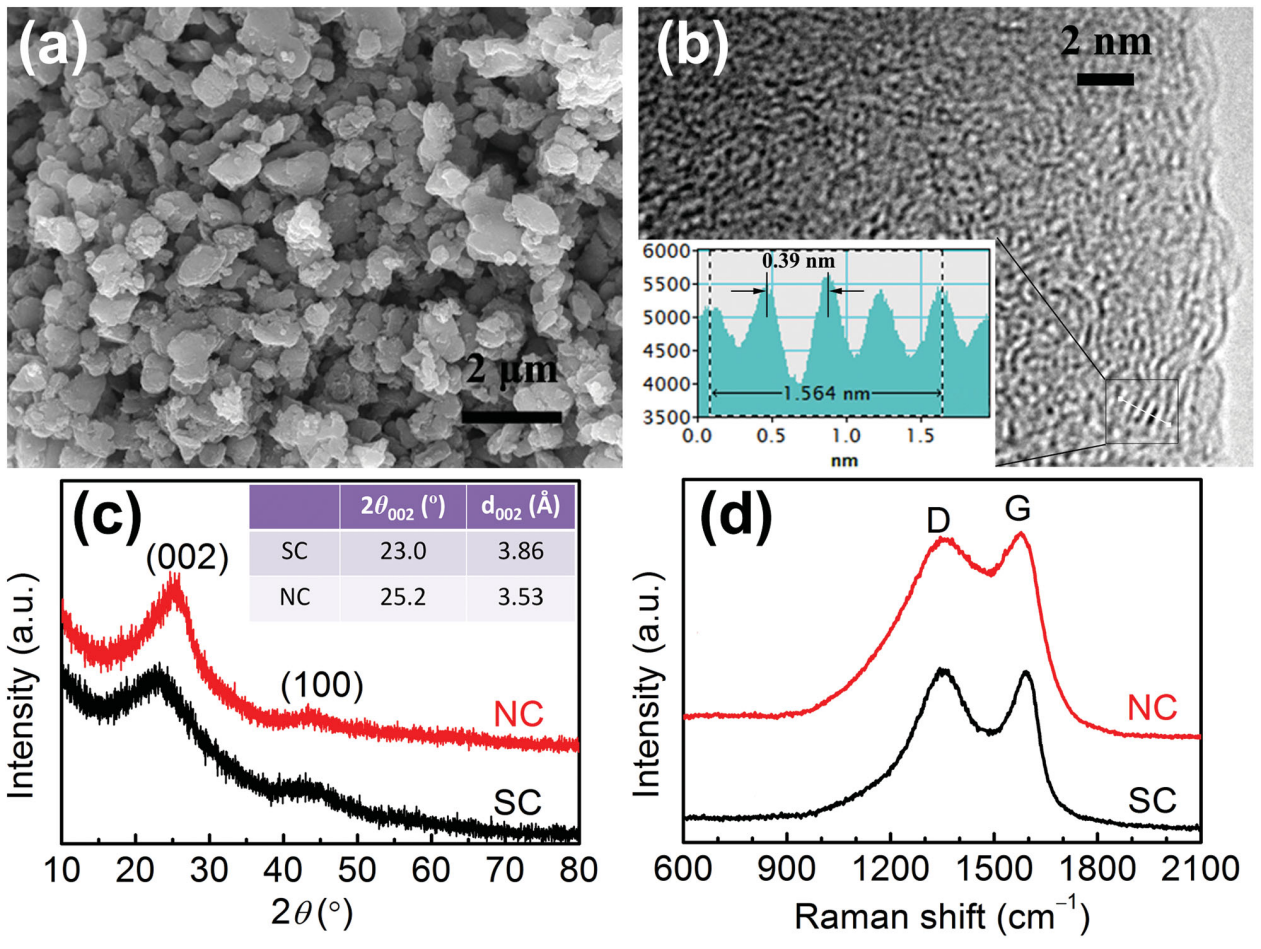
a) SEM image of SC; b) HRTEM image of SC; c) XRD patterns of SC and NC; and d) Raman spectra of SC and NC. Inset of (b) shows the corresponding intensity profile for the line scan across the lattice fringes.

The microstructures of SC and NC were further investigated by high‐resolution transmission electron microscopy (HRTEM). From the HRTEM image in Figure 1b, amorphous nature of SC and graphite microcrystallites with an average interlayer distance of 0.39 nm can be observed on the edge of the particle. While for NC, the observed interlayer distance is only ≈0.36 nm (Figure S 2 b, Supporting Information), much smaller than SC. Such results show that the introduction of S atoms is more effective in enlarging the interlayer distance of carbon than N atoms, which is consistent with the previous report.^20^ This is because the covalent radius of S is much larger than those of C and N, and consequently, the interlayer distance increases with the incorporation of sulfur atoms into the carbon structure. Compared with the reported two‐step oxidation–reduction process,^15^ S‐doping provides a facile route to produce carbons with enlarged interlayer distance.

**Figure 2:**
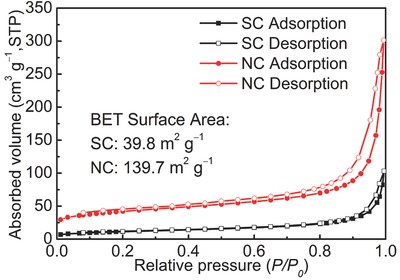
Nitrogen adsorption–desorption isotherms of SC and NC.

The difference in interlayer distance between SC and NC could also be confirmed by X‐ray diffraction (XRD) patterns. As shown in Figure 1c, both of them present two weak, broad peaks of (002) and (100) diffractions, indicative of amorphous nature. The (002) peaks for SC and NC are centered, respectively, at around 23.0 and 25.2°, corresponding to interlayer distances (*d*_002_, calculated based on the Bragg's law) of 3.86 and 3.53 Å, which agree with the HRTEM observations.

In addition to the interlayer distance, the Na^+^‐ion storage capacity can also be affected by the graphitization degree of carbon. Raman spectroscopy analysis was employed to investigate the graphitization degrees of SC and NC. Both of the spectra in Figure 1d reveal broad disorder‐induced D‐bands at ≈1350 cm^−1^ and in‐plane vibrational G‐bands at ≈1590 cm^−1^.^6^

The porosity was examined by nitrogen adsorption–desorption measurement for SC and NC. As shown in **Figure** **2**, both of the isotherms can be classified to type II isotherms, revealing the nonporous or macroporous nature of SC and NC.^21^ However, due to the larger particle size, the calculated Brunauer–Emmett–Teller (BET) specific surface area of bulk‐sized SC is 39.8 m^2^ g^−1^, much smaller than that of nanosheet‐like NC (139.7 m^2^ g^−1^). Such results demonstrate that increasing particle size is efficient to reduce the specific surface area of carbon. The decreased surface area can suppress the formation of solid electrolyte interphase (SEI) layer and thus increase the initial Coulombic efficiency of the carbonaceous anodes for SIBs.

The CHNSO elemental analysis for PEDOT, PPy, SC, and NC was performed on an elemental analyzer. As seen in **Table** **1**, the S content in SC is as high as 15.17 wt% and the N content in NC is 12.14 wt%. Such high‐level heteroatoms can change the microstructure of the carbon and hence influence the electrochemical performance.^22^ Here, a tiny amount of S detected in PPy and NC is originated from the oxidant and dopant during the chemical polymerization process.^23^ The results agree well with the energy‐dispersive X‐ray (EDX) spectroscopy in Figure S6 (Supporting Information).

**Table 1:** Elemental compositions of PEDOT, PPy, SC, and NC

Sample	Chemical composition [wt%]				
	C	O	H	S	N
PEDOT	50.72	27.02	3.82	18.68	–
SC	77.05	5.59	2.74	15.17	–
PPy	56.84	20.13	4.21	2.47	16.12
NC	66.19	17.64	2.72	1.16	12.14

X‐ray photoelectron spectroscopy (XPS) was employed to detect the nature of sulfur species at the surface of PEDOT and SC. As shown in **Figure** **3**a, the S2p spectrum of PEDOT can be fitted into four peaks: two major peaks at 163.9 and 165.0 eV are assigned to S 2p_3/2_ and S 2p_1/2_ spectra for the —C—S—C— covalent bond of thiophene‐type sulfur owning to the spin–orbit splitting,^24^ while two minor peaks at higher energy (166.0 and 168.3 eV) are consistent with –C–S(O)_2_–C– sulfone bridges.^25^ After carbonization, the peak intensity of the –C–S(O)_2_ –C– bond becomes very low (Figure 3b), which is consistent with the elemental analysis results, indicating that most of the oxidized sulfur species were decomposed during the thermal treatment and sulfur is mainly doped at the edges and defects in the form of thiophene‐type structure in SC.^26^ Based on the XPS analysis, a possible structure of SC containing S functional groups is given in the inset of Figure 3b.

**Figure 3:**
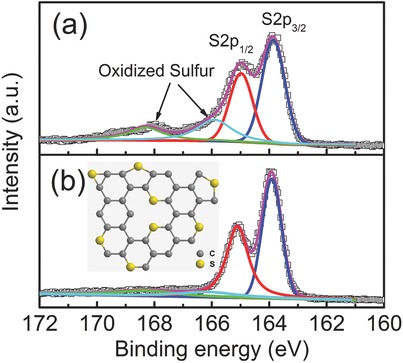
High‐resolution S2p XPS spectra of a) PEDOT and b) SC. Inset of (b) shows the schematic model of the sulfur functional groups in SC.

The Na^+^‐ion storage properties of SC and NC were evaluated by galvanostatic discharge/charge tests in a potential window of 2.0–0.01 V versus Na^+^/Na. Different from the reported typical two‐region discharge curves for hard carbons, which showed both sloping region (2.0–0.15 V) of the intercalated Na^+^ ions and plateau region (≈0.1 V) of the absorbed Na^+^ ions,^13^, ^27^ the SC only shows sloping region in the discharge curves (**Figure** **4**a), indicating that most of the capacity for SC is contributed by the insertion of Na^+^ ions into the parallel or nearly parallel carbon layers. The unconspicuous sloping plateaus centered at ≈1.3 V in the discharge and ≈1.7 V in the charge (Figure 4a) can be addressed by the pseudocapacitance and/or the chemisorption of Na^+^ ions caused by the sulfur functional groups in SC, which may also contribute to the capacity. Similar sloping discharge curves (Figure S7a, Supporting Information) are also observed in NC which specific surface area is much higher than SC. The absence of the plateau region in low voltage reveals that the contribution to the total capacity from the adsorption of Na^+^ ions onto the nanoporosity or surface of the carbon is almost negligible, demonstrating that the capacity of such carbons cannot be enhanced via simply increasing their porosity.^28^ It should also be mentioned that although the sloping charge curves of SC could reduce the energy density of the full cell, the absence of low‐voltage plateau is helpful to avoid the safety problem caused by deposition of Na metal on the surface of the hard carbon anode, which is also a major concern for the application of SIBs.[[qv: 10c]]

**Figure 4:**
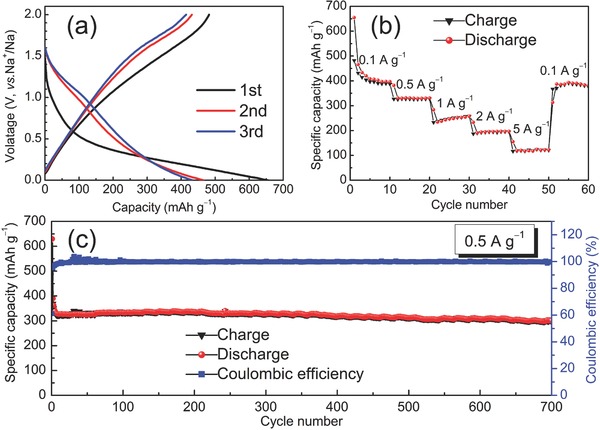
Electrochemical performances of SC as anode for SIBs: a) discharge/charge curves at 0.1 A g^−1^, b) capacity over cycling at different rates, and c) cyclability and Coulombic efficiency at 0.5 A g^−1^.

As anode for SIBs, SC exhibits high capacity and excellent rate performances. As shown in Figure 4b, SC delivers a high initial reversible specific capacity of 482.1 mAh g^−1^ with a Coulombic efficiency of 73.6% at a current density of 0.1 A g^−1^, much higher than those of most reported carbonaceous anodes for SIBs.^5^, ^8^,s[[qv: 10a]] The irreversible capacity (172.9 mAh g^−1^) of SC in the first cycles, which is originated from the decomposition of electrolyte associated with the formation of SEI on the surface of carbons, is much smaller than that of NC (266.7 mAh g^−1^, Figure S7b, Supporting Information), confirming that reducing specific surface area is effective to mitigate the SEI formation and thus to enhance the initial Coulombic efficiency of the carbonaceous anodes. With increasing the current density, stable capacities of 327.8, 242.0, 192.5, and 119.5 mAh g^−1^ can be attained at 0.5, 1, 2, and 5 A g^−1^, respectively. When the current density is tuned back to 0.1 A g^−1^, a charge capacity is recovered up to 367.0 mAh g^−1^.

The long‐term cycling performance of SC was evaluated at a current density of 0.5 A g^−1^. As shown in Figure 4c, the reversible capacity is as high as 384.5 mAh g^−1^ in the first cycle and stabilized to 322.0 mAh g^−1^ at the tenth cycle. Even after 700 cycles, a capacity is still maintained at 303.2 mAh g^−1^. Compared to the stabilized capacity, the corresponding capacity retention is as high as 94.2% over 700 cycles, indicating that the enlarged interlayer distance of SC guarantees the structural stability during the repeated sodiation/desodiation processes. As a comparison, the NC with higher specific surface area and smaller interlayer distance only exhibits a capacity of 116.9 mAh g^−1^ after 500 cycles at 0.5 A g^−1^ (Figure S7c, Supporting Information). Obviously, the SC displays superior Na^+^‐ion storage performance to the N‐doped one.

The schematic diagrams in **Figure** **5** illustrate the Na^+^‐ion intercalation in graphite, NC, and SC. The Na^+^‐ion storage performances of the carbons are greatly influenced by their interlayer distances. Due to the small interlayer distance (3.35 Å), it is difficult for graphite to electrochemically intercalate Na^+^ ions. For the amorphous hard carbons with lower graphite degree and larger interlayer distance, such as the PPy‐derived NC, the electrochemical intercalation of Na^+^ ions becomes available but is still limited since the average interlayer distances are not large enough. The situation is different for SC. When large‐sized S atoms are introduced into carbon, the interlayer distance of carbon is greatly enlarged to 3.86 Å, which can not only accommodate more Na^+^ ions but also facilitate the Na^+^‐ion insertion and extraction. In addition, the pseudocapacitance and/or chemisorption of Na^+^ ions originated from the introduction of the N and S containing functional groups might also contribute to the total capacity of the carbons. Using an approach developed by Dunn and co‐workers,^29^ the calculated capacitive contributions for the total charge storage of SC and NC are, respectively, 35.4% and 28.8% at 0.1 mV s^−1^ (see the detailed calculation in the Supporting Information, Figures S8 and S9).

**Figure 5:**
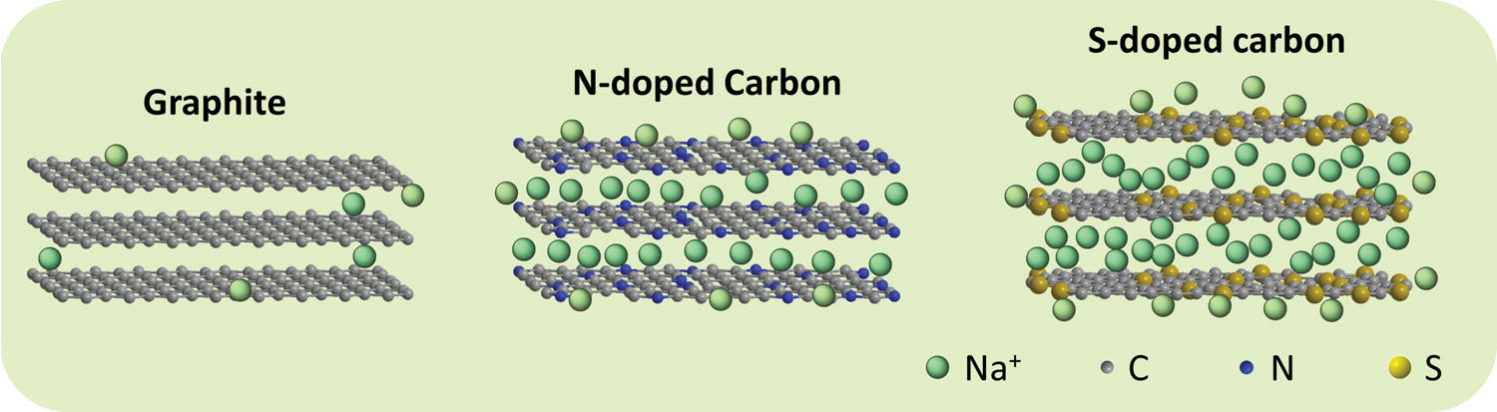
Schematic diagrams for Na^+^‐ion storage in graphite, N‐doped carbon, and S‐doped carbon.

In summary, for the first time, we have investigated the Na^+^‐ion storage properties of SC. The obtained SC shows bulk‐sized morphology with a specific surface area of 39.8 m^2^ g^−1^. With the introduction of high‐level (15.17 wt%) large‐sized S atoms into carbon, the SC displays enlarged interlayer distance (3.86 Å). As anode for SIBs, SC shows high initial Coulombic efficiency (73.6%), excellent rate capability, and stable cyclability. The specific capacity is as high as 303.2 mAh g^−1^ even after 700 cycles at 0.5 A g^−1^. The present work indicates that large interlayer distance and low specific surface area are desirable to achieve high performance for the carbonaceous anode materials of SIBs. S‐doping in carbons is an effective strategy to obtain high‐performance SIBs for future commercial application.

## Experimental Section

*Synthesis of PEDOT and PPy Precursors*: The precursors (PEDOT and PPy) were synthesized by chemical oxidation polymerization with ammonium persulfate (APS) as oxidant and dodecylbenzene sulfonic acid (DBSA) as dopant. Typically, the monomer (EDOT or Py, 1 mL) and DBSA (5 g) were added in deionized (DI) water (50 mL) and stirred for 30 min. Then, the oxidant (APS, 4 g) dissolved in 10 mL DI water was added to the above mixture solution. By being stirred for 24 h at room temperature, a precipitate was formed. The precipitate was washed with ethanol and DI water till the filtrate became colorless and neutral. Finally, the product (PEDOT or PPy) was dried overnight at 80 °C in an oven.

*Synthesis of SC and NC*: PEDOT (or PPy) was heated up to 700 °C with a heating rate of 3 °C min^−1^ and kept for 2 h under an argon atmosphere to form SC (or NC).

*Characterization*: The morphology was observed with SEM (SIRION200) coupled with an EDX (Oxford Instrument). TEM observation was carried out on a JEOL 2100 microscope. The phase was checked by XRD (PANalytical B.V., Holland) using Cu Kα radiation at *λ* = 1.54 Å, Raman spectra (LabRAM HR800), and Fourier transform infrared spectrometer (Bruker, VERTEX 70). The CHNSO elemental analysis was performed with an elemental analyzer (Vario Micro cube). XPS measurements were carried out on a VG MultiLab 2000 system with a monochromatic Al Kα X‐ray source (ThermoVG Scientific). Nitrogen adsorption and desorption isotherms were determined by nitrogen physisorption at 77 K on a Micromeritics ASAP 2020 analyzer.

*Electrochemical Measurements*: The electrochemical performances of the SC and NC were measured with 2032 coin cells. Na metal was used as anode, NaClO_4_ (1 mol L^−1^) in a mixture of ethylene carbonate and propylene carbonate (3:1 by volume) as electrolyte, and glass fiber (GF/D, Whatman) as separator. The working electrode was made of SC or NC (85 wt%), super P (5 wt%), and polyvinylidene fluoride (10 wt%) slurry coated onto a copper foil substrate. Before testing, it was dried in a vacuum oven at 80 °C for 24 h. The galvanostatic discharge/charge tests were carried out between 2.0–0.01 V versus Na^+^/Na on a Land CT2001A (China).

## Supporting information

As a service to our authors and readers, this journal provides supporting information supplied by the authors. Such materials are peer reviewed and may be re‐organized for online delivery, but are not copy‐edited or typeset. Technical support issues arising from supporting information (other than missing files) should be addressed to the authors.

Supplementary
