# Supplementary material for: Sulfur‐Doped Carbon with Enlarged Interlayer Distance as a High‐Performance Anode Material for Sodium‐Ion Batteries
Source: Adv Sci (Weinh). 2015 Aug 25;2(12):n/a. doi: 10.1002/advs.201500195 (PMC5049484; doi:10.1002/advs.201500195)
Supplement: Supplementary file 1 — Supplementary [file ADVS-2-0k-s001.pdf]

Copyright WILEY-VCH Verlag GmbH & Co. KGaA, 69469 Weinheim, Germany, 2015.

Supporting Information

for *Adv. Sci.*, DOI: 10.1002/advs. 201500195

Sulfur-Doped Carbon with Enlarged Interlayer Distance as a High-Performance Anode Material for Sodium-Ion Batteries

*Long Qie, Weimin Chen, Xiaoqin Xiong, Chenchen Hu, Feng Zou, Pei Hu, and Yunhui Huang**

Copyright WILEY-VCH Verlag GmbH & Co. KGaA, 69469 Weinheim, Germany, 2015.

Supporting Information

Sulfur-Doped Carbon with Enlarged Interlayer Distance as a High-Performance Anode Material for Sodium-Ion Batteries

*Long Qie, Weimin Chen, Xiaoqin Xiong, Chenchen Hu, Feng Zou, Pei Hu and Yunhui Huang**

Prof. Y. H. Huang, Dr. L. Qie, Dr. W. Chen, X. Xiong, C. Hu, Dr. F. Zou, P. Hu
Key Laboratory for Advanced Battery Materials and System, Ministry of Education; School of Materials Science and Engineering, Huazhong University of Science and Technology, Wuhan, Hubei 430074, China
E-mail: huangyh@hust.edu.cn

Keywords: Sodium ion battery; Anode; Doped Carbon; Electrochemical performance

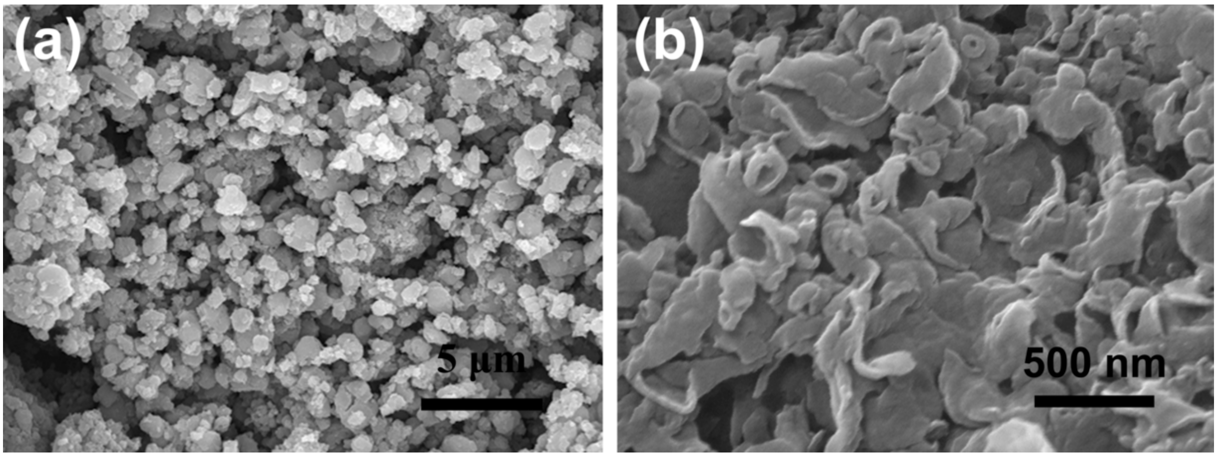

Figure S1. SEM images of (a) PEDOT and (b) PPy precursors.

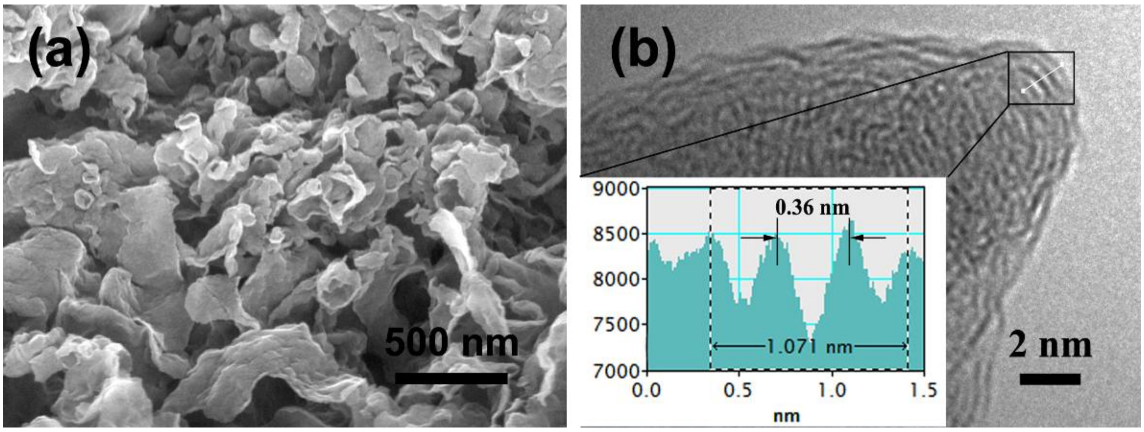

Figure S2 (a) SEM image of SC, and (b) HRTEM image of SC. Inset of (b) shows the corresponding intensity profile for the line scan across the lattice fringes.

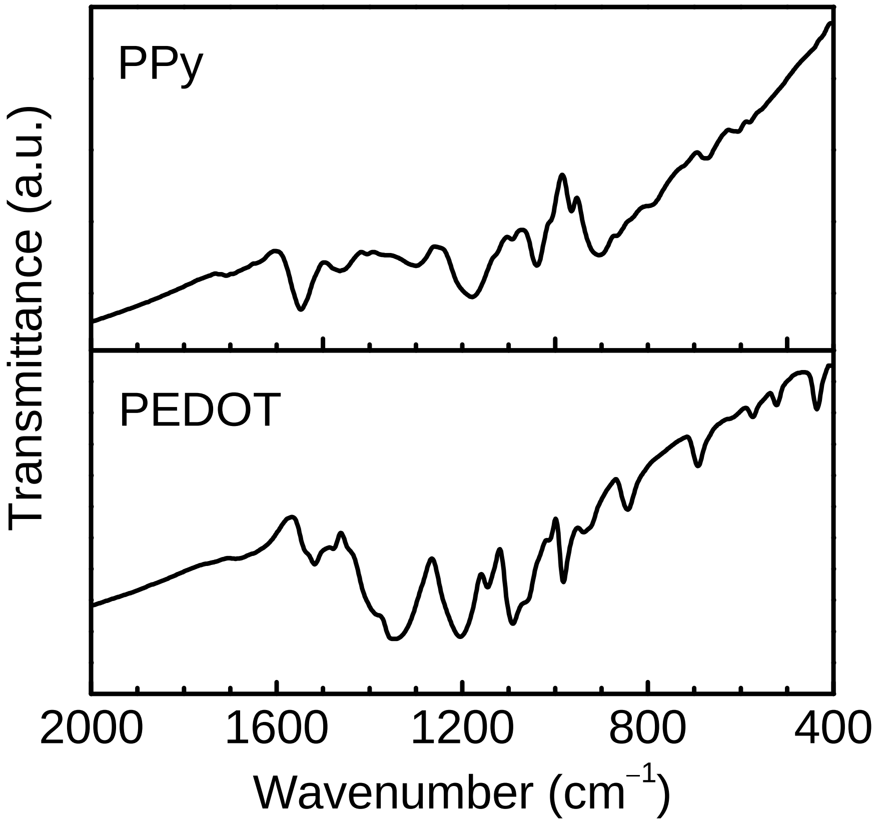

Figure S3. FTIR of PEDOT and PPy precursors.

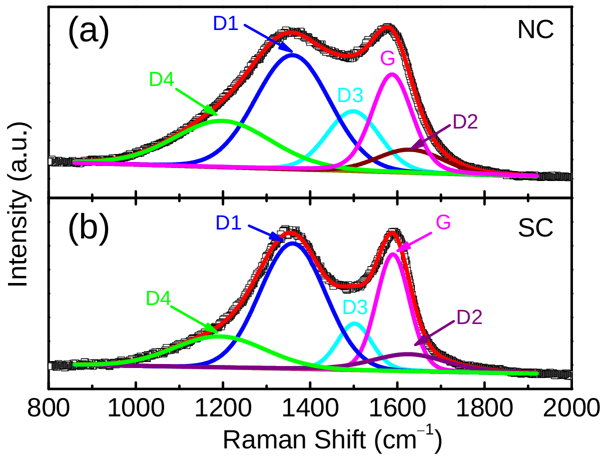

Figure S4 Curve fitting for the Raman spectra of SC and NC.

The Raman spectra of SC and NC could be deconvolved into five bands (G, D1, D2, D3, and D4), respectively, at about 1585, 1345, 1625, 1500, and 1200 cm^{-1}.[1] The calculated D1/G band intensity (peak area) ratios for NC and SC are, respectively, 1.99 and 2.03, indicating the similar graphite degrees of NC and SC.

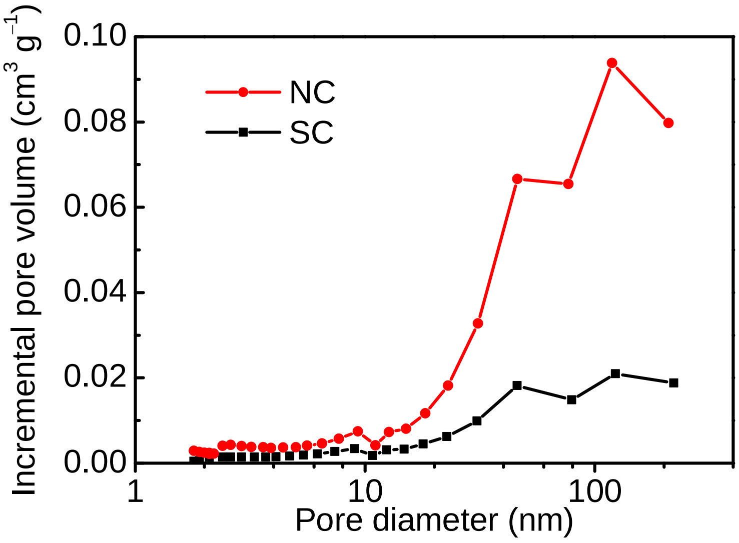

Figure S5 Pore size distributions of SC and NC.

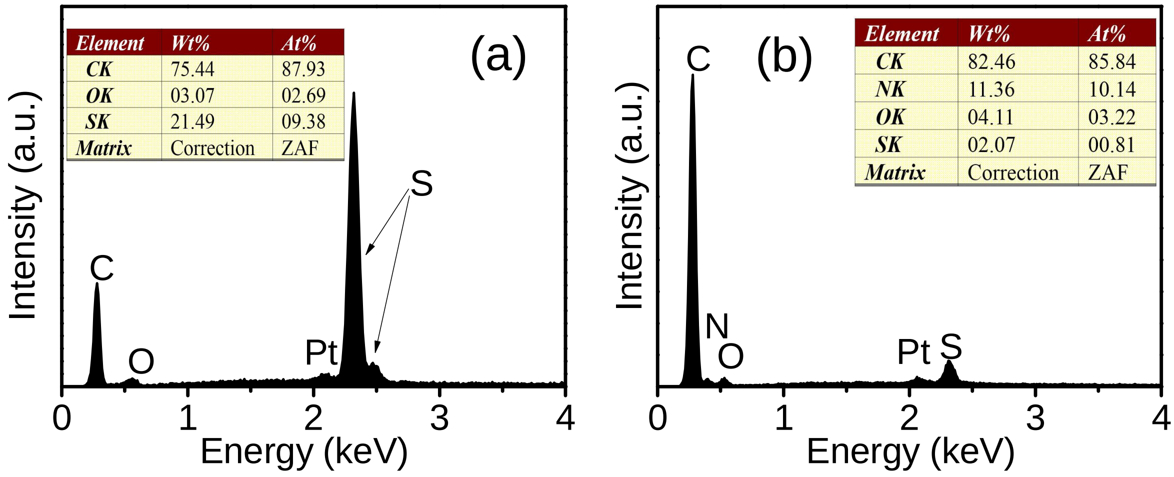

Figure S6. EDX spectra of (a) SC and (b) NC.

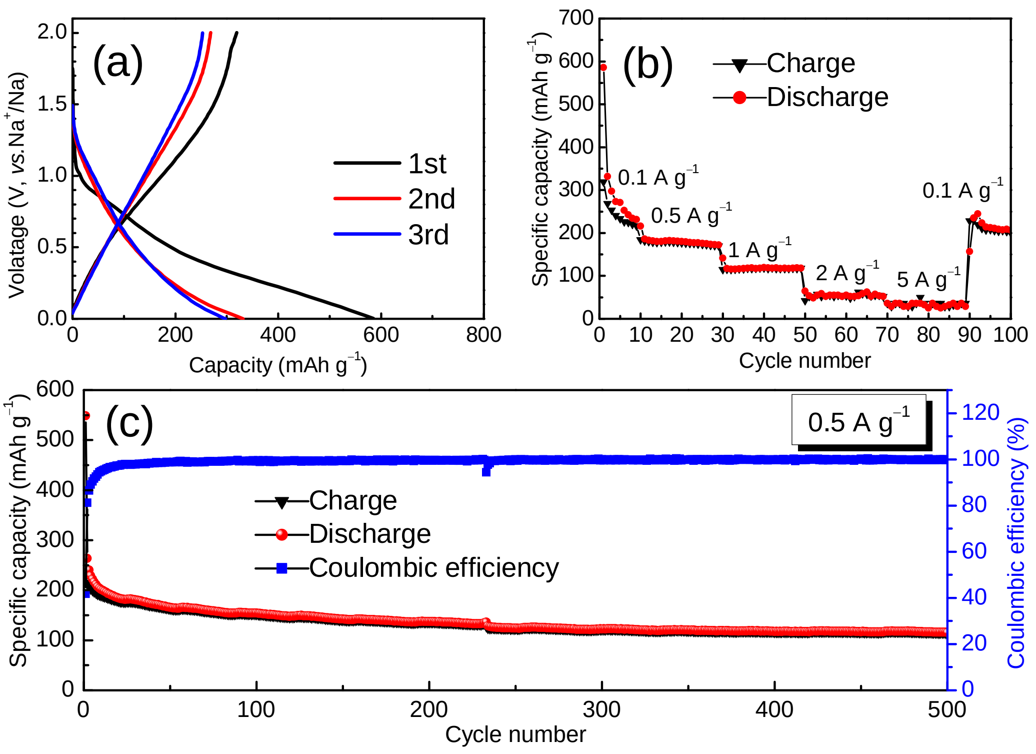

Figure S7. Electrochemical performances of NC as anode for SIBs: (a) Discharge/charge curves at 0.1 A g^{-1}, (b) capacity over cycling at different rates, and (c) cyclability and Coulombic efficiency at 0.5 A g^{-1}.

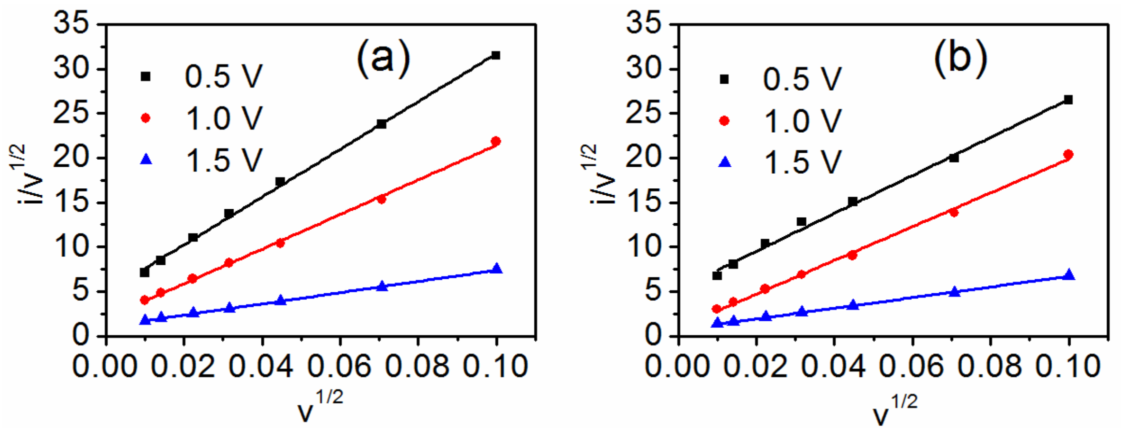

Figuer S8 The polts of $i/v^{1/2}$ *vs.* $v^{1/2}$ at 0.5, 1.0 and 1.5 V for (a) SC and (b) NC.

To determine the contributions of capacitance and intercalated Na^+ ions, cyclic voltammetry (CV) curves with multi-scan rates from 0.1 to 10 mV s^{-1} were carried out and the data were used to quantify the capacitive contribution based on the previous approach developed by Dunn *et al.*[2] The measured current of CV curves could be separated into two components: (1) the capacitive contribution and (2) the contribution from diffusion-controlled current of Na^+ insertion. The current response (i) at a fixed potential (V) can be represented by the following equation:

$$i(V) = k_1 v + k_2 v^{1/2}$$

Where $k_1 v$ represents the surface capacitive charge, and $k_2 v^{1/2}$ stands for the contribution of diffusion-controlled charge. The linear plot of $i(V)/v^{1/2}$ as a function of $v^{1/2}$ could be used to determine the slope (k_1) and intercept (k_2) (**Figure S8**), and contributions of capacitance and intercalated Na^+ ions at a certain voltage could be estimated with the value of k_1 and k_2.

The calculated capacitive contributions for the total charge storage of SC and NC are, respectively, 35.4% and 28.8% at 0.1 mV s^{-1} (**Figure S9**).

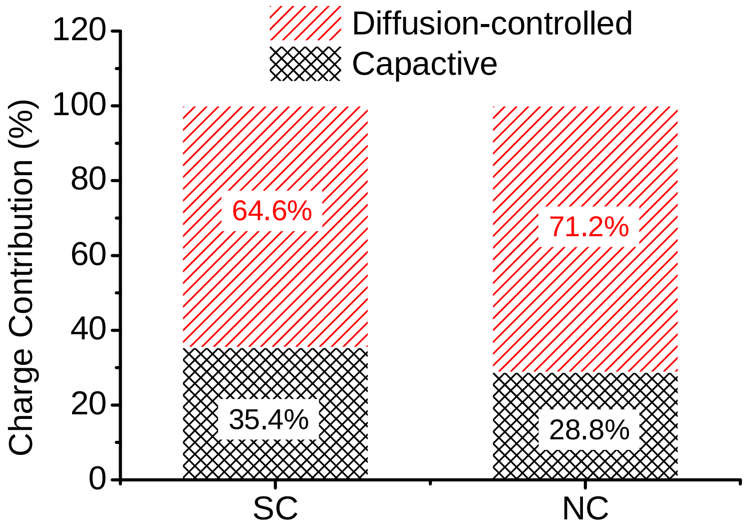

Figure S9 Charge contributions from capacitance and diffusion-controlled process for SC and NC at a scan rate of 0.1 mV s^{-1}.

[1] A. Sadezky, H. Muckenhuber, H. Grothe, R. Niessner, U. Pöschl, *Carbon* **2005**, *43*, 1731.

[2] J. Wang, J. Polleux, J. Lim, B. Dunn, *J. Phys. Chem. C* **2007**, *111*, 14925.
